# Mucosal Delivery of Cannabidiol: Influence of Vehicles and Enhancers

**DOI:** 10.3390/pharmaceutics14081687

**Published:** 2022-08-13

**Authors:** Peera Tabboon, Thaned Pongjanyakul, Ekapol Limpongsa, Napaphak Jaipakdee

**Affiliations:** 1 Division of Pharmaceutical Technology, Faculty of Pharmaceutical Sciences, Khon Kaen University, Khon Kaen 40002, Thailand; 2 Center for Research and Development of Herbal Health Products, Khon Kaen University, Khon Kaen 40002, Thailand; 3 College of Pharmacy, Rangsit University, Pathum Thani 12000, Thailand

**Keywords:** cannabidiol, cannabinoids, permeation, deposition, fatty acids, terpenes

## Abstract

In this study, the mucosal permeation and deposition of cannabidiol (CBD) with neat and binary vehicles were investigated. Permeation experiments were performed using static diffusion cells coupled with fresh porcine esophageal mucosa. The CBD–vehicle solutions were applied at a fixed dose (~5 mg/cm^2^), and the corresponding permeation parameters were calculated. In neat vehicles, the permeation flux (*J_ss_*) ranged from 0.89 ± 0.15 to 179.81 ± 23.46 µg·cm^−2^·h^−1^, while the CBD deposition ranged from 11.5 ± 1.8 to 538.3 ± 105.3 μg·cm^−2^. Propylene glycol (PG) and diethylene glycol monoethyl ether (DEGEE) yielded the highest permeability (*P_s_*) and CBD deposition, while medium-chain triglycerides (MCT) yielded the lowest *P_s_* and deposition. This was due to the difference in apparent partition coefficient (*K*), which is related to the solubility of CBD in the vehicle. The PG:DEGEE binary vehicle boosted *J_ss_* (1.5–1.6 fold) and deposition (2.0–2.7 folds) significantly, compared to neat DEGEE. The combination of DEGEE with MCT dramatically enhanced *J_ss_* (11–44 fold) and deposition (1.6–4.7 fold). The addition of lipophilic enhancers, laurocapram, and oleic acid, to PG:DEGEE and DEGEE:MCT vehicles significantly reduced *J_ss_* (0.3–0.7 fold) and deposition (0.4–0.8 fold) while nerolidol had no effect. These permeation reductions were found to be related to modification of the *K* and/or diffusivity values. This study provides useful basic information for the development of CBD formulations intended for transmucosal delivery.

## 1. Introduction

Cannabidiol (CBD), a non-psychotropic cannabinoid present in high concentrations in the cannabis plant, has attracted substantial interest due to its broad pharmacological activities (e.g., antiepileptic, anticonvulsant, antipsychotic, antianxiety, and neuroprotective). In particular, it has been investigated extensively for its antiepileptic properties, as well as its feasible applications in the treatment of neuropsychiatric disorders, inflammatory diseases, pain perception, cancer, and other symptoms [1,2,3]. Concerning the Biopharmaceutics Drug Disposition Classification System (BDDCS), CBD is classified as a Class II compound due to its high permeability, low water solubility, and being eliminated extensively via metabolism [3]. From a physicochemical point of view, CBD is classified as a BCS Class II compound. It exhibits low water solubility (<5 µg/mL) and high lipophilicity with the calculated log K_ow_ of 8 and pKa of 9.3 [3,4]. However, the extensive first-pass metabolism and variable absorption restrict the bioavailability of CBD to approximately 6% when administered orally [5]. Therefore, alternative administration routes with suitable drug delivery systems or preparations for CBD delivery have been investigated [1,3,6].

Buccal transmucosal delivery has been recognized as a promising and efficient alternative for systemic drug delivery. The buccal route offers numerous advantages, in comparison to the peroral route, including: (i) the ability to circumvent the hepatic first-pass effect and deterioration in the gastrointestinal tract; (ii) the ability to provide a rapid onset of action, which is associated with the relatively permeable and highly vascularized buccal mucosa, especially in the sub-lingual region; (iii) good patient compliance; and (iv) ease of self-medication. In addition, buccal administration is also regarded as a useful route for palliative care [7,8,9]. Nevertheless, the barrier properties of mucosal tissue still limit the fraction of drug permeating into and through the buccal mucosa. An effective strategy to overcome this mucosal barrier effect is the utilization of chemical permeation enhancers which are able to partition into and interplay with mucosal constituents, resulting in barrier property impairment and/or modification of the solubility of a permeant compound in the biological membrane. Numerous substances have been examined as permeation enhancers, in order to improve the flux/absorption of drugs through the mucosa, such as alcohols/glycols, terpenes, laurocapram, oleic acid, and so on. The enhancer of choice and its permeation potency rely on the physico-chemical properties of the permeant, as well as those of the vehicles and the vehicle composition [10,11].

It is well known that the composition of vehicles could modulate the permeation flux and lag time. Regarding the polarity of the available liquid vehicles that function as permeation enhancers, they can be categorized as hydrophilic, polar (e.g., ethanol; propylene glycol, PG; diethylene glycol monoethyl ether, DEGEE) or lipophilic, non-polar, (e.g., isopropyl myristate; medium-chain triglycerides, MCT). The combination of these liquid vehicles may offer a synergistic response upon mucosal delivery, principally when the vehicles having different permeation enhancement mechanisms are combined, and provide a potential ability to reduce mucosal irritation. Enhanced transdermal and mucosal drug delivery has been observed with mixtures of two or three vehicles, as the binary or ternary systems, compared with neat vehicles [12,13,14,15].

Oral transmucosal administration of cannabinoid solutions, as oromucosal sprays or drops, has been shown to be an efficient means for cannabinoid systemic delivery [16]. The vehicles used in this dosage form function as solvents/co-solvents, as well as permeation enhancers. However, this preparation generally involves the use of a high concentration of ethanol, which has been associated with a bad taste and mucosal irritation. Non-alcoholic preparations, thus, are interesting alternatives. To date, a limited number of publications have reported on the mucosal permeation and deposition behaviors of CBD solutions. Therefore, the object of this study is to assess the mucosal permeation and deposition performances of CBD with various vehicles. A range of neat vehicles, including MCT, DEGEE, PG, and polyethylene glycol 300 (PEG 300), as well as binary combinations of these liquids, were investigated. The influence of combined enhancers, including laurocapram, nerolidol, and oleic acid was also assessed.

## 2. Materials and Methods

### 2.1. Materials

Cannabidiol (CBD, CBD isolate, 99%) was kindly provided by the Medicinal Cannabis Research Institute, College of Pharmacy, Rangsit University (Thailand). Medium-chain triglyceride, NF (MCT) was sourced from Spectrum Chemical Mfg. Corp. (New Brunswick, NJ, USA). Absolute ethanol and oleic acid were sourced from QRëC (Auckland, New Zealand). Diethylene glycol monoethyl ether (DEGEE) was sourced from Beijing Solarbio Science & Technology Co., Ltd. (Beijing, China). Propylene glycol (PG, Kollisolv^®^ PG) and polyethylene glycol 300 (PEG 300, Kollisolv^®^ PEG 300) were sourced from BASF SE (Ludwigshafen, Germany). Laurocapram and nerolidol were provided by J&H Chemicals (Hangzhou, China) and Sigma-Aldrich (Missouri, MO, USA), respectively. Formic acid was supplied from KemAus (Cherrybrook, Australia). HPLC grade methanol and acetonitrile were supplied by Fisher^®^ Scientific (Loungborough, UK). All chemicals were used as received.

### 2.2. CBD Solubility

The equilibrium saturated concentration of CBD in the neat vehicles—namely MCT, DEGEE, PG, and PEG 300—was determined using the shake-flask solubility assay method. In brief, 1 g of CBD was added to 1 mL of each test vehicle in a well-closed amber vial. The CBD–vehicle mixture was sonicated for 1 h (ultrasonicator Model LUC-405, Daihan Labtech Co. Ltd., Namyangju-si, Korea) and then shaken continuously at 37 °C for 24  h (shaking water bath Model LSB-030S, Daihan Labtech Co. Ltd., Namyangju-si, Korea). The resulting supersaturated CBD solution was filtered through 0.45 μm nylon syringe filter media with polypropylene housing. This solution was diluted suitably and quantitative analysis of the CBD was conducted through HPLC assay. Experiments were performed in triplicate.

### 2.3. Vehicle Uptake of Mucosa

The uptake capacities of the neat vehicles (MCT, DEGEE, PG, and PEG 300) and binary vehicles (25:75 PG:DEGEE and 50:50 PG:DEGEE; as well as 25:75 DEGEE:MCT, 50:50 DEGEE:MCT, and 25:75 DEGEE:MCT) with or without combined enhancers (laurocapram, oleic acid, and nerolidol) into the mucosa were determined gravimetrically, using the method described by Watkinson with modification [17]. Porcine esophageal mucosa was utilized as a surrogate of non-keratinized buccal mucosa [18]. The esophageal mucosa was prepared, as reported previously [19], cut into 2 × 2 cm^2^ samples, and oven-dried at 60 °C for 24 h (drying oven SLW 115 STD, POL-EKO-APARATURA sp.j., Wodzislaw Slaski, Poland) to a constant weight. The dried mucosa was weighed (*W*_0_) and soaked in 5 mL of the investigated vehicle at 37 °C for 8 h with gentle shaking (shaking water bath Model LSB-030S, Daihan Labtech Co. Ltd., Namyangju-si, Korea). After 8 h, the soaked mucosa was collected, blotted dry with an absorbent tissue, and then re-weighed (*W_t_*). The increase in mucosa weight after vehicle uptake, calculated by Equation (1), was used to determine the uptake capacity. Each experiment was carried out in triplicate.

(1) $$Vehicle\;uptake\;capacity\;\left(\%\right)=\frac{\left(W_{t}-W_{0}\right)}{W_{0}}\times 100$$

### 2.4. Ex Vivo Permeation

#### 2.4.1. CBD Solution Preparation

A finite concentration of CBD solution was prepared by dissolving a known amount of CBD in the required volume of a vehicle to yield a 2.5% *w*/*v* solution. A range of CBD solutions in neat (MCT, DEGEE, PG, and PEG 300) and binary (PG:DEGEE (25:75 and 50:50% *v*/*v*) and DEGEE:MCT (25:75, 50:50, and 25:75% *v*/*v*)) vehicles, as well as binary vehicles with 5% *w*/*v* of the combined enhancers (laurocapram, oleic acid, and nerolidol), were prepared. Each CBD solution was freshly prepared on the day of the permeation and deposition experiments.

#### 2.4.2. Mucosal Membrane Preparation

The esophageal mucosa was prepared using a previously reported protocol [19]. The whole porcine esophagus from a freshly slaughtered adult pig was received as a waste product from a local abattoir (Khon Kaen, Thailand). The mucosal layer of the lumen was carefully removed from the underneath muscularis layers. The harvested esophagus lumen was thoroughly rinsed with phosphate-buffered saline and an incision was made longitudinally to yield a mucosa sheet. The resulted mucosa, with a thickness of 627 ± 9 μm, was cut into 2.5 × 2.5 cm^2^ pieces, rinsed with phosphate-buffered saline to remove any loose debris, and used immediately.

#### 2.4.3. Permeation Study

The mucosal permeation of CBD was studied ex vivo using modified Franz diffusion cells with a diffusional area of 2.01 cm^2^ and receptor compartment volume of 14.0 ± 0.2 mL. Fresh esophageal mucosa, with the mucosa side oriented toward the donor compartment, was mounted on each diffusion cell. The receiver medium was 50% *v*/*v* ethanol in deionized water (pH 5.8). The mucosa equipped within the diffusion cells, with the receiver medium in the receiver side and 1.5 mL of pH 6.8 simulated saliva fluid (SSF) contained in the donor side, was permitted to equilibrate for 15 min. To start the permeation, pH 6.8 SSF in the donor side was removed and the mucosa was blotted with an absorbent tissue. Then, the freshly prepared CBD solution (400 μL) was pipetted and placed into the donor compartment, which was then occluded with parafilm. The receptor compartment was controlled at 37 °C under continuous stirring using a magnetic stirrer at 80 rpm (digital magnetic stirrer, VELP SCIENTIFICA, Usmate Velate, Italy). At predetermined times (1, 2, 3, 4, 5, 6, 7, and 8 h), 800 µL of receiver medium was collected and substituted with 800 µL of pre-warmed fresh medium. The collected samples were vacuum-dried using a vacuum concentrator (SpeedVac SPD300DDA, Thermo Scientific, Waltham, MA, USA) set at 45 °C. The residue was reconstituted with 0.2 mL of methanol before being analyzed for the permeated amount of CBD by HPLC assay.

#### 2.4.4. Permeation Data Analysis

The cumulative mass of CBD permeated per unit area was calculated and plotted against time. The steady-state flux (*J_ss_*) was determined from the steady-state portion of each permeation curve as per Equation (2). Lag time (*T_lag_*) was accessed by extrapolating the linear fraction of the permeation curve to the time axis. The permeability coefficient of mucosa (*P_s_*), the partition coefficient of CBD between mucosa and vehicle (*K*), and the apparent diffusivity through the mucosa (*D_ss_*) are defined as Equations (2) and (3), respectively. *Q*_8*h*_, the cumulative CBD permeated at 8 h, was calculated using Equation (4).

(2) $$J_{ss}=P_{s}\cdot C_{d}=\frac{K\cdot D_{ss}\cdot C_{d}}{L}=\frac{\Delta Q_{t}}{\Delta t\cdot A}$$

(3) $$T_{lag}=\frac{L^{2}}{6D_{ss}}$$

(4) $$Q_{t}=\frac{C_{t}\cdot V+\left(\sum_{t-1}^{t}C_{t-1}\right)\cdot V_{w}}{A}$$

(5) $$Enhancement\;ratio\;\left(ER\right)=\frac{J_{ss}\;of\;tested\;vehicle}{J_{ss}\;of\;the\;control}$$

where *C_d_* is the CBD concentration in the donor compartment (2.5% *w*/*v*) and *L* is the mucosa thickness. *Q_t_* refers to the cumulative permeated amount of CBD, while *C_t_* is the CBD concentration at the time *t*. *C_t_*_–1_ denotes the CBD concentration at the previous time point. *V* is the total volume of receiver medium, while *V_w_* refers to the withdrawal volume of receiver medium at each time point. *A* is the permeation surface area. ∆*Q_t_* is the difference in Q_t_ between time points and ∆*t* is the time difference [11,20,21,22].

### 2.5. Ex Vivo Deposition

After the permeation assessment (8 h), the remaining donor solution was discarded. The mucosa surface was patted dry using an absorbent tissue, and then detached from the diffusion cell. The mucosa, held in a slightly tilted position, was rinsed with deionized water (1 mL, 3 times), followed by methanol (1 mL, 3 times) and deionized water (1 mL, 3 times), in order to wash off the CBD remaining on its surface. After patting with an absorbent tissue, the diffusional area of mucosa exposed to CBD solution was isolated, cut into tiny pieces using surgical scissors, and placed into a 15 mL centrifuge tube. Extraction of CBD deposited in the mucosa was performed by vortexing with 2 mL of methanol for 10 min, followed by sonicating for 5 min. The supernatant was collected after centrifugation. The extraction was carried out three times. All extraction supernatants were combined, then vacuum-dried (at 45 °C) using a vacuum concentrator (SpeedVac™ SPD300DDA, Thermo Scientific, Waltham, MA USA). The obtained residue was reconstituted with 2 mL methanol, filtered through a 0.45 µm syringe filter, and analyzed for CBD content using HPLC. The deposition was calculated in terms of the amount per surface area (µg·cm^−2^).

### 2.6. HPLC Assay

An Agilent 1260 Infinity HPLC System (Germany) equipped with an autosampler and a diode array detector set to 220 nm was used for CBD analysis. Chromatographic separation was carried out at 45 °C using a reverse-phase ZORBAX Eclipse Plus C18 column (4.6 mm × 100 mm, 3.5 μm) with an isocratic system at a flow rate of 1.5 mL/min. The mobile phase was composed of 0.1% formic acid in acetonitrile/water (70/30 by volume). The injection volume was set at 20 μL. The retention time of CBD was shown at the peak area of 6.02 min with a 10 min run-time. The chromatographic method exhibited excellent linearity over a concentration range of 2–80 μg/mL with correlation coefficient values higher than 0.999.

### 2.7. Statistical Analysis

Experiment results are expressed as mean ± SD. One-way analysis of variance (ANOVA) with Tukey’s post-hoc test, performed using the SPSS Statistics for Windows software (Version 17.0, Released 2008, Chicago, IL, USA: SPSS Inc.), was conducted for statistical analysis. The statistical significance level was set at 95% (*p* < 0.05).

## 3. Results and Discussion

In the current investigation, ex vivo mucosal permeation and deposition of CBD from various vehicles, applied at a fixed concentration (2.5% *w*/*v*) and dose (~5 mg/cm^2^), were determined. The neat and binary systems of four vehicles—namely, MCT as a lipophilic vehicle, and DEGEE, PG, and PEG 300 as hydrophilic vehicles—were investigated. The effects of combined enhancers, including laurocapram, oleic acid, and nerolidol, were also examined. These vehicles and enhancers were selected based on their proven feasibility in increasing the permeation of a number of compounds through mucosa and skin [10,11,12,13,14,15,21,23,24], as well as their frequency of use in pharmaceutical preparations.

### 3.1. Effect of Neat Vehicles on CBD Solubility, Permeation and Deposition

#### 3.1.1. CBD Solubility

The solubility of CBD in neat vehicles, together with their respective properties reported in the literature [23,25,26,27,28], is tabulated in Table 1. The CBD solubility values were ranked as follows: PEG 300~MCT > DEGEE > PG (*p* < 0.05). Regarding the present results, CBD was considered very soluble in MCT and PEG 300, and freely soluble in DEGEE and PG.

The solubility is normally regulated by the chemical properties of the solute and liquid solvent. It is well known that a solute will dissolve in a solvent having similar molecular structure, size, and polarity (like dissolves like theory) [32]. CBD is considered a lipophilic compound with a log K_ow_ of 8 [4] and the calculated Hansen solubility parameter of CBD is 22.8 MPa^1/2^ [6]. Thus, the higher solubility of CBD in MCT may be due to their similar polarities. With regards to the dielectric constant, MCT is considered a non-polar solvent which, thus, may facilitate a considerable lipophilic interaction between CBD and MCT (induced dipole-induced dipole interaction) [32]. In the case of PEG 300, its higher CBD solubility could be related to the similarity in their Hansen solubility parameters. The Hansen solubility parameter is the well-known numerical constant that is generally used to describe the solvency property of compounds, as well as to predict the miscibility or solubility of solute–solvent systems. It is well known that a solute exhibits its maximum solubility in a liquid solvent having a similar solubility parameter value. Nevertheless, dissolution is a spontaneous, yet complex process, involving the breaking of solute–solute and solvent–solvent bonds while forming the solute–solvent interaction. The use of a single parameter is generally insufficient to predict and describe the solubility of any solute–solvent system, particularly those possessing complex molecular structures.

#### 3.1.2. CBD Permeation and Deposition

The permeation of CBD from the investigated vehicles was determined using modified Franz diffusion cells coupled with the fresh porcine esophageal mucosa. This mucosa has been suggested as an alternative permeability barrier to buccal mucosa, due to their similarities in structure and permeability characteristics [18,33]. Moreover, porcine esophageal mucosa can be prepared easily and efficiently, as intact and even-thickness mucosa can be obtained with a high yield [33]. The CBD solution with various vehicles was applied at a fixed concentration (2.5% *w*/*v*) and dose (~5 mg/cm^2^). To achieve the sink condition, 50% *v*/*v* ethanol in deionized water was utilized as a receiver medium [6,34]. The solubility of CBD in 50% ethanol has been found to be adequate to maintain sink conditions throughout permeation investigations [6]; however, it should be noted that ethanol may influence the integrity of the mucosa. It is well-known that ethanol has the ability to improve the permeability of drugs across biological membranes [10,11]. In the case of percutaneous permeation, a study using FTIR has revealed that ethanol influenced the intercellular lipids in the stratum corneum. At present, no FTIR study has yet proven the ability of ethanol to extract buccal mucosal lipids. It is still doubtful whether this intercellular lipid extraction caused by ethanol—especially at a high concentration of ethanol—occurs in the buccal mucosa due to the less-ordered intercellular lipid domains than in the stratum corneum. Veuillez et al. [35] have reported that buffered ethanolic solution (30:70 ethanol:phosphate buffer pH 7.4) did little or no damage to the structure of the buccal epithelial mucosa. It has also been claimed that ethanol had no noticeable effect on the structure of the porcine buccal mucosa [36]. Nevertheless, as 50% *v*/*v* ethanol was used for all permeation investigations in this study, the effect of this liquid on the mucosa was assumed to be identical. In this way, the effects of different vehicles and enhancers could be comparatively assessed. Similarly, 50% ethanolic deionized water has previously been used as a receiver liquid in permeation investigations of very lipophilic compounds, including cannabinoids [6,34,36,37].

**Figure 1:**
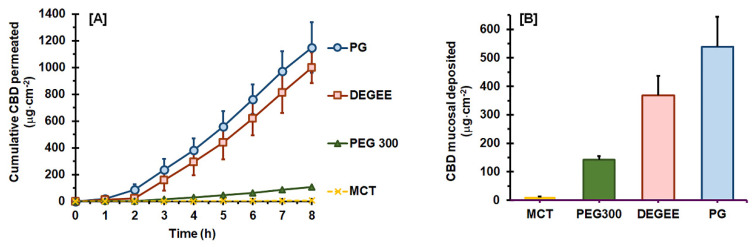
Ex vivo permeation profiles of CBD through porcine mucosa from various neat vehicles (**A**) and the mucosal deposition amount of CBD after permeation (**B**) (mean ± SD, *n* = 4).

The mucosal permeation profiles of CBD with the neat vehicles are presented in Figure 1A, while their corresponding permeation parameters are listed in Table 2. The mucosal permeation flux ranged from 0.89 ± 0.15 to 179.81 ± 23.46 µg·cm^−2^·h^−1^. The corresponding permeability (*P_s_*) and the partition coefficient (*K*) values were ranked in the order of PG~DEGEE > PEG 300~MCT (*p* < 0.05). As a result, PG delivered the highest cumulative CBD permeated at 8 h; that is, approximately 23.1 ± 3.8% of the applied dose, which was comparable to that with DEGEE (20.1 ± 2.3% of the applied dose) (*p* > 0.05). All investigated neat vehicles exhibited a comparable lag time within the range of 1.7–2.2 h (*p* > 0.05). These were caused by the equivalent apparent diffusivity through the mucosa (*D_ss_*) of CBD obtained from neat PG, DEGEE, PEG 300, and MCT (*p* > 0.05).

The deposition mass of CBD in the permeated mucosa, in terms of μg per permeated area, is illustrated in Figure 1B. The highest CBD deposition was found with neat PG (538.3 ± 105.3 μg·cm^−2^), followed by DEGEE (368.0 ± 68.4 μg·cm^−2^), PEG 300 (538.3 ± 105.3 μg·cm^−2^), and MCT (11.5 ± 1.8 μg·cm^−2^), respectively. In terms of the percentage of the applied dose, only 0.2 ± 0.0% of the applied CBD was mucosally deposited with neat MCT, while 10.8 ± 2.1% and 7.4 ± 1.4% of the applied CBD was found in the mucosal membrane with neat PG and DEGEE, respectively.

It was noted that the deposition mass of CBD in hydrophilic vehicles seems to be related to the uptake of vehicles into the dry mucosal membrane. As shown in Table 2, the vehicle uptake capacity into dry mucosa presented in the order of PG > MCT > DEGEE > PEG 300 (*p* < 0.05). Nevertheless, the uptake performance of vehicles may differ between fresh and dry mucosa, especially for MCT. The extent of MCT uptake into the fresh mucosa is probably much lower than that into the dry one, due to the immiscibility between MCT and the hydrating medium. This might have caused the irrelevant relationship between MCT uptake into the dry mucosa and CBD deposition amount.

The lower CBD permeation from PEG-based compared with PG-based vehicles was in line with the report of Casiraghi et al. [6]. This report has recently demonstrated that the percutaneous permeation flux, cumulative permeated amount at 24 h, and deposition amount of CBD from 80:20 PEG 400:water vehicle were significantly less than those obtained with 80:20 PG:water vehicle. The higher permeation flux and permeability of CBD from hydrophilic than from lipophilic vehicles were also in line with previous reports [6,38,39]. Stinchcomb et al. [38] have investigated the ex vivo permeation of cannabinoids—namely, CBD, Δ^8^-tetrahydrocannabinol, and cannabinol—through human skin. They found that a 4:5:4 PG:water:ethanol vehicle yielded a higher permeation flux, permeability, and skin deposition of CBD than with mineral oil. With the hydrophilic vehicle, the mean percutaneous permeation flux was 1.47 ± 0.23 µg·cm^−2^·h^−1^ with no lag time, while mineral oil exhibited a CBD flux of 0.23 ± 0.06 µg·cm^−2^·h^−1^ with *T_lag_* of 10.5 ± 5.8 h. Casiraghi et al. [6] have recently reported the permeation parameters of CBD obtained from an ex vivo study using human skin. The results indicated that an 80:20 PG:water vehicle exhibited a higher flux with shorter *T_lag_* (1.06 ± 0.34 µg·cm^−2^·h^−1^, 1.95 ± 0.79 h) than with virgin olive oil (0.02 ± 0.01 µg·cm^−2^·h^−1^, >7 h), which seemed to be related to the higher solubility of CBD in the virgin olive oil (>300 mg/mL) than in the PG:water vehicles (16.23 ± 0.51 mg/mL).

Permucosal permeation refers to the passage through the mucosal membrane, involving diffusion of the permeant from the vehicle to the mucosal surface, then partitioning into and diffusing across mucosal layers. The mucosal permeation pathways can be either transcellular (intracellular) and/or paracellular (intercellular) [40]. With regard to its physico-chemical properties, the transport of CBD through the mucosa is likely to occur through a paracellular pathway, where it diffuses through the intercellular lipid components of the intercellular spaces between the cells [41]. As presented in Figure 1 and Table 2, the permeation *J_ss_* and *P_s_* of CBD varied, in accordance with the neat vehicle. As described in Equation (2), *P_s_* is a composite variable that includes *D_ss_*, *K*, and mucosal thickness (*L*). As the *L* value was controlled and all vehicles exhibited comparable *D_ss_*, the lower CBD permeation flux with neat PEG 300 and MCT was therefore attributable to the lower *K* values caused by the lower thermodynamic activity of CBD in these vehicles, as compared to those in neat PG and DEGEE. It is well known that the partition of permeant into the biological membrane is driven by thermodynamic activity, which decreases with the solubility of the permeant in the vehicle [42]. The partition coefficient, *K,* is a measure of the relative affinity of CBD for mucosa and vehicle. The twice-higher solubility of CBD in PEG 300 and MCT than in PG and DEGEE caused a decrease in *K* and, thus, *J_ss_*. The lower partition coefficients and permeation flux, in relation to the high affinity or solubility of permeant in the vehicle, were in agreement with other reports [13,15,43]. Caon et al. [44] have reported that PEG 400 retarded the permeability of donepezil across the buccal mucosa. The permeation of naratriptan through porcine buccal mucosa has been found to be the lowest with neat PEG 400, while neat DEGEE yielded the greatest flux [15]. In a later study, Alomrani et al. [12] found that neat PEG 400 provided the lowest percutaneous permeation flux of piperine compared to the rest of the investigated vehicles (i.e., ethanol, PG, oleic acid, and water).

The highest permeation of CBD from neat PG may also be associated with the mucosal uptake of PG in a sufficient quantity, which may alter the solubilizing properties of the mucosa with respect to CBD, promoting the partition of CBD into the mucosal membrane [24,45]. The capacity of PG to “carry” the permeate as it diffuses across the membrane has been suggested. Numerous studies have correlated its permeation potency to its ability to permeate into and deposit in the biological membrane [46,47,48,49]. Moreover, PG can affect lipid bilayers by interacting with the polar head groups of lipid bilayers, modifying the solubility of the biological membrane and increasing the permeant partitioning into it [24,50]. The highest mucosal uptake of PG also resulted in the highest CBD mucosal deposition. PG is one of the most frequently used glycols in topical, transdermal and mucosal preparations, due to its good safety profile. The feasibility of using PG for the skin and mucosal delivery enhancement of CBD has been recently reported [6,39,51].

For DEGEE, it has been proposed that the major mechanism of permeation improvement involves the modification of permeant solubility in the biological membrane and improvement of the partitioning of permeants (*K* parameter). Harrison et al. [52] have claimed that the permeation enhancement of cyanophenol from DEGEE was mainly due to the effect of DEGEE on the solubility, instead of diffusivity in the membrane. Puglia and Bonina [53] have suggested that the enhancement effect of DEGEE on the permeation of atenolol from emulsion preparations was related to the increase in the apparent membrane vehicle partition coefficient, rather than the diffusion coefficient. A positive correlation between the permeation degree of DEGEE and the permeant has been reported [54,55]. The prompt uptake of DEGEE into the biological membrane and its accumulation in it indicate a ‘pull’ effect, assisting in permeation and deposition improvement. It has also been reported that DEGEE causes intercellular lipid fluidization without altering the bilayer structure [23,55,56]. DEGEE has been examined as a potential permeation enhancer for the transdermal delivery of CBD [39,57]. The permeation of DEGEE through biological membranes has been demonstrated in a number of in vitro studies [15,47,55,58]. DEGEE, an ether alcohol with an approved safety profile, is a common liquid vehicle used in topical, transdermal, and mucosal preparations, as well as oral and parenteral pharmaceutical formulations. It offers advantages over other vehicles, as it is clear (transparent) non-volatile, very low viscosity, nearly odorless, and miscible with polar and non-polar liquids. Investigations of up to 100 different permeants have indicated the efficacy of DEGEE in terms of solubilization, permeation flux, and/or deposition in the biological membrane [23]. Iliopoulos et al. [49] have described that neat DEGEE had a comparable capacity to deliver niacinamide into the skin as neat PG. These neat vehicles demonstrated higher percutaneous permeation of niacinamide, compared to that obtained with the other neat vehicles; namely, 1–2 hexanediol, 1–2 pentanediol, 1–5 pentanediol, 1–3 butanediol, glycerol, and dimethyl isosorbide. The potential of DEGEE as a permeation enhancer for buccal delivery has been demonstrated [15,59].

### 3.2. Effect of Binary Vehicles on Permeation Flux and Deposition

#### 3.2.1. PG–DEGEE Binary Systems

**Figure 2:**
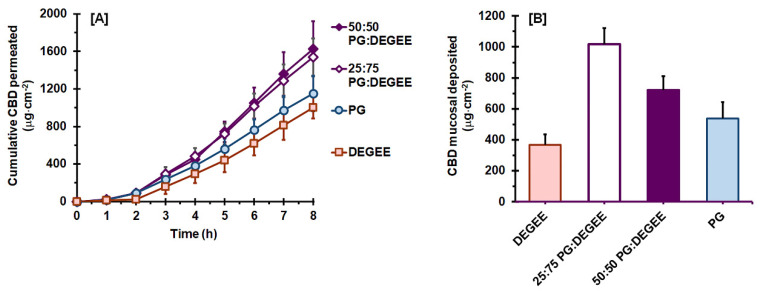
Ex vivo permeation profiles (**A**) and mucosal deposition amount (**B**) of CBD with the PG:DEGEE binary vehicle, when compared with neat DEGEE and PG (mean ± SD, *n* = 4).

Regarding the permeation behaviors of CBD from neat vehicles, the two best vehicles—namely, PG and DEGEE—were combined and investigated. The permeation profiles of CBD from PG:DEGEE binary systems, as compared with those of neat PG and DEGEE, are presented in Figure 2A. It can be clearly seen that the addition of PG promoted the mucosal permeation of CBD, compared to when using neat DEGEE. As listed in Table 3, the permeation *J_ss_*, *P_s_*, and *Q*_8*h*_ of the PG:DEGEE binary systems were higher than those of neat DEGEE (*p* < 0.05), yet comparable to those of neat PG (*p* > 0.05). *T_lag_* and *D_ss_* were not affected by the addition of PG, as these values for the PG:DEGEE systems were comparable to those of neat vehicles (*p* > 0.05). It should be noted that there was no significant difference in the permeation parameters, as well as the CBD deposition, for the 25:75 and 50:50 PG:DEGEE binary systems (*p* > 0.05). This is in line with the report of Kung et al. [14] who demonstrated that 25:75 PG:DEGEE and 50:50 PG:DEGEE vehicles provided no significant difference in the permeation performance of methadone.

The deposition mass of CBD in the permeated mucosa (Figure 2B) from PG:DEGEE systems was also significantly increased, compared to those of the neat vehicles. The CBD deposition values were 1017.4 ± 104.1 μg·cm^−2^ (20.3 ± 2.1% of the applied dose) and 720.9 ± 91.1 μg·cm^−2^ (14.4 ± 1.8% of the applied dose) for 25:75 and 50:50 PG:DEGEE systems, respectively. These results suggest that PG:DEGEE binary systems appear to be promising vehicles to provide a CBD mucosal reservoir. The capacity of the buccal mucosa to function as a CBD reservoir has been suggested in an experiment performed in vivo by Itin et al. [41]. This finding demonstrated that the accumulation of CBD in the oral mucosa through the administration of CBD in a 1:1 ethanol:PG solution using a mucoadhesive “shell” lengthened the CBD release into systemic circulation, for up to 4 h following removal of the device.

When compared to neat DEGEE, the permeation flux of the investigated PG:DEGEE systems was improved by 1.5–1.6 times. After 8 h, approximately 30.9–32.8% of the applied dose of CBD in the PG:DEGEE systems could be delivered through the mucosal membrane, and approximately 14.4–20.3% of the applied dose was deposited in the mucosal membrane. According to the permeation parameters, it can be seen that this permeation enhancement was related to the increase in the *P_s_* values of PG:DEGEE systems, associated with the *K* value increment. The significant increase in the CBD deposition from PG:DEGEE systems—namely, 2.8-fold and 2.0-fold increment for 25:75 and 50:50 PG: DEGEE systems, respectively, compared with neat DEGEE—may be associated with the increased vehicle uptake capacity. As listed in Table 3, the PG:DEGEE systems had a significantly higher vehicle uptake than neat DEGEE (*p* < 0.05). The presence of PG facilitated the uptake of DEGEE into the mucosal membrane, which may modulate the mucosal environment adequately for CBD partitioning and, thus, permeation as well as deposition [14,58]. This is in agreement with the report of Kung et al. [14], in which PG was shown to exhibit a ‘pull’ impact, facilitating higher DEGEE uptake into the skin. This was claimed to contribute to increased drug solubility in intracellular lipid, drive the partitioning of the permeant into the skin, and increase percutaneous permeation. The absence of a pronounced synergistic effect on CBD percutaneous permeation by PG:DEGEE systems, compared to neat PG vehicle, was in agreement with a recent report investigating the percutaneous permeation of CBD [39]. According to this report, the permeation flux and *T_lag_* of CBD from 60:40 PG:DEGEE were comparable to those of neat PG vehicles. Additionally, 60:40 PG:DEGEE exhibited a significantly lower amount of CBD deposited in the epidermis layer.

#### 3.2.2. DEGEE–MCT Binary Systems

**Figure 3:**
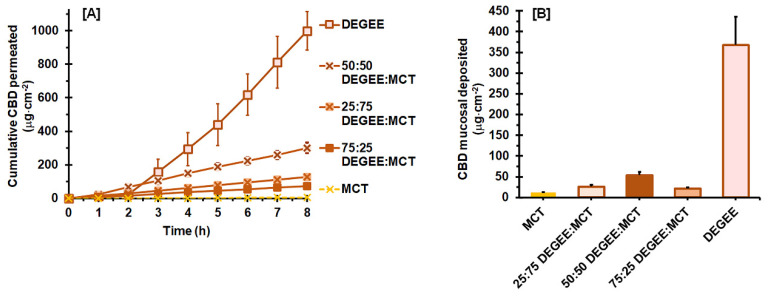
Ex vivo permeation profiles (**A**) and mucosal deposition amount (**B**) of CBD with the DEGEE:MCT binary vehicle systems, when compared with neat MCT and DEGEE (mean ± SD, *n*= 4).

Next, the influence of hydrophilic vehicles on the mucosal permeability and deposition of CBD with an MCT-based vehicle was investigated; however, amongst the hydrophilic vehicles investigated, DEGEE was the only vehicle that was completely miscible with MCT. Therefore, binary DEGEE–MCT systems were prepared at different ratios, and assessed for their effect on the permeation and deposition performance of CBD. As presented in Figure 3A, it is obvious that the addition of DEGEE to MCT promoted the CBD permeation. On the other hand, the presence of MCT caused a dramatic decrease in CBD permeation, compared to the neat DEGEE vehicle.

The effect of DEGEE concentration on the mucosal permeation parameters of MCT-based systems is presented in Table 4. The values of neat MCT and DEGEE are also included, for comparative purposes. When compared with neat MCT, the addition of DEGEE to the MCT vehicle enhanced the permeation *J_ss_* by 17.7, 43.8, and 10.9 folds for 25:75, 50:50, and 75:25 DEGEE:MCT, respectively. The *J_ss_* and *P_s_* of DEGEE:MCT binary systems were ranked as follows: 50:50 DEGEE:MCT > 25:75 DEGEE:MCT > 75:25 DEGEE:MCT > MCT (*p* < 0.05). However, when compared with neat DEGEE, the addition of MCT resulted in a worse permeation effect, where *J_ss_* and *P_s_* were decreased by 10.0, 4.2, and 17.2 folds for 25:75, 50:50, and 75:25 DEGEE:MCT, respectively. Furthermore, their *Q_8h_* values diminished by 7.9, 3.4, and 13.7 fold for 25:75, 50:50, and 75:25 DEGEE:MCT, respectively.

The DEGEE:MCT binary systems significantly shortened the *T_lag_* to the range of 0.23 ± 0.05 to 0.26 ± 0.07 h, whereas those of the neat vehicles were within 2.09 ± 0.31 to 2.17 ± 0.18 h. This was associated with the higher apparent *D_ss_* obtained from DEGEE:MCT binary systems, compared to that of MCT (*p* < 0.05). It should be noted that there was no significant difference in the *T_lag_* (0.23 ± 0.05 to 0.26 ± 0.07 h) and apparent *D_ss_* (2.623 ± 0.425 ×10^−3^ to 2.984 ± 0.835 × 10^−3^ cm^2^·h^−1^) values between the DEGEE:MCT systems (*p* > 0.05).

The greater *J_ss_* and *P_s_* values of the DEGEE:MCT systems were related to the increased *K* and *D_ss_* values. The maximum *J_ss_* was observed with 50:50 DEGEE:MCT, where 5.8% of the applied CBD dose was permeated after 8 h. This may be related to the higher thermodynamic activity of CBD in 50:50 DEGEE:MCT. The solubility of CBD in this vehicle mixture was expected to be lower than that of MCT, due to the contribution of DEGEE. This caused a shift in partitioning between the mucosal membrane and the vehicle, as indicated by the highest *K* value of the 50:50 DEGEE:MCT system. Additionally, the presence of MCT was found to promote the uptake of DEGEE into the mucosal membrane. This further increases the “carrier-solvent” effect of DEGEE to pull the CBD together down into the mucosal membrane. Such synergistic enhancement in the mucosal permeation of MCT:DEGEE systems is in line with the results of Sattar and Lane [15]. According to their study, although neat MCT did not result in a high value of permeation flux, it yielded the shortest *T_lag_* of naratriptan permeation through porcine buccal mucosa, as compared to the investigated hydrophilic vehicles. The binary systems of MCT with DEGEE could significantly promote the rate and extent of naratriptan permeation, compared with neat DEGEE, while not affecting the deposition amount. The synergistic enhancement mechanism obtained for MCT:DEGEE systems was suggested to be due to the thermodynamic activity modification caused by the lower solubility of naratriptan in the binary vehicle.

It is interesting to note that a further increase in the DEGEE concentration in MCT-base vehicle to 75% decreased the *J_ss_* of CBD. The *Q_8h_* values for 75:25 and 25:75 DEGEE:MCT systems were comparable (*p* > 0.05). This was attributed to the lower partition coefficient of 75:25 DEGEE:MCT, compared to 25:75 DEGEE:MCT and 50:50 DEGEE:MCT. From this, it can be assumed that the affinity of CBD to the vehicle might increase and, so, the partitioning of CBD into the mucosa could be reduced in the case of 75:25 DEGEE:MCT vehicle.

Figure 3B illustrates the mucosal deposition amount of CBD after permeation from DEGEE:MCT systems, which ranged from 21.5 ± 2.9 to 53.9 ± 8.0 μg·cm^−2^, compared with 11.5 ± 1.8 μg·cm^−2^ in neat MCT. The addition of 25%, 50%, and 75% of DEGEE to the MCT vehicle raised the CBD deposition amount by 2.3, 4.7, and 1.6 folds, respectively. Similar to the permeation results, the CBD deposition amount from MCT-based vehicle increased with DEGEE concentration up to 50%, yet a further increase to 75% DEGEE decreased the deposition. Nevertheless, when compared with neat DEGEE, the presence of 25%, 50%, and 75% MCT greatly reduced the CBD deposition, compared to neat DEGEE vehicle, by 14.1, 6.8, and 17.1 fold, respectively. It is interesting to note that the uptake values of vehicles into dry mucosa values were not correlated with either the deposition or permeation of CBD from the MCT-based vehicles. As shown in Table 4, the vehicle uptake capacity into dry mucosa was in the order of neat MCT~25:75 DEGEE:MCT > 50:50 DEGEE:MCT ~ 75:25 DEGEE:MCT (*p* < 0.05).

MCT is a mixture of triglycerides of saturated fatty acids; mainly caprylic (C_8_) acid and capric (C_10_) acid. It is believed that the permeation enhancement of lipophilic components (e.g., vegetable oil, fatty acids/alcohols, glycerides, isopropyl myristate), including MCT, is related to the similar polarity of these components to that of lipids in the biological membrane. In addition to the occlusive effect, these lipophilic vehicles might incorporate themselves into lipid bilayers and distort the organization of lipids [6,56,60]. The feasibility of MCT as a potential excipient in buccal formulations has been recently revealed [15].

The synergistic action of binary systems of hydrophilic and lipophilic vehicles on the percutaneous permeation of numerous permeants has been reported [49,55,61,62]. These finite-dose investigations demonstrated that permeation improvement was attained by blending lipophilic vehicles (e.g., isopropyl myristate, oleic acid, linolenic acid, and propylene glycol monolaurate) with PG as a hydrophilic vehicle. The ability of an ethanol:tricaprylin (40:60) binary vehicle to increase the percutaneous flux while shortening the lag time of numerous permeants, including salicyluric acid, salicylic acid, alclofenac, ketoprofen, ibuprofen, and tegafur, has been reported. The mechanistic enhancement of this binary vehicle was described as being due to the effects on the *D_ss_* value increment by tricaprylin and the *K* value by ethanol [61,62]. Nevertheless, evidence of such binary systems behaving as permeation retardants has also been reported. It was recently found that the percutaneous permeation flux of CBD from PG-based vehicles decreased by approximately 4.3 times when a lipophilic vehicle, 10% isopropyl myristate, was added. Moreover, the *T_lag_* and deposition amount of CBD found in the epidermis layer were also diminished with the addition of this lipophilic vehicle [39]. The retardation effect of isopropyl myristate was found to be related to its high lipophilicity (log P of 6.5) which might reduce the ability of CBD to partition into the skin.

### 3.3. Effect of a Combined Permeation Enhancer on Permeation Flux and Deposition

It is well known that different permeation enhancers affect the permeation of permeants distinctively, considering their different mechanisms of action. In this study, the CBD permeation-enhancing effect of three different compounds—namely, laurocapram, nerolidol, and oleic acid—at 5% *w*/*v* concentration in hydrophilic- and lipophilic-based vehicles were investigated. The hydrophilic- and lipophilic-based vehicles investigated were 25:75 PG:DEGEE and 25:75 DEGEE:MCT, respectively.

**Figure 4:**
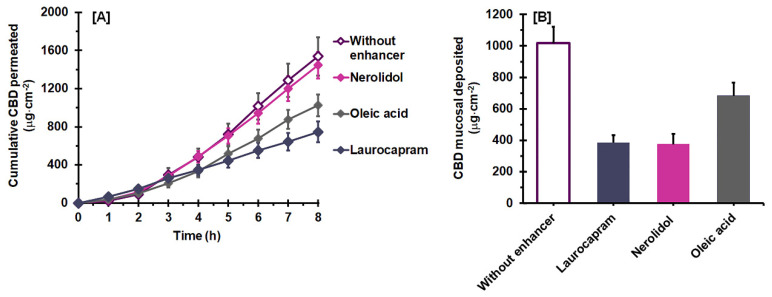
Effect of combined enhancers on ex vivo CBD permeation (**A**) and deposition (**B**) with the 25:75 PG:DEGEE binary vehicle (mean ± SD, *n* = 4).

Figure 4A demonstrates the influence of a combined enhancer on the mucosal permeation behavior of CBD with a hydrophilic vehicle, 25:75 PG:DEGEE. It is clear that nerolidol had no influence on the CBD permeation performance with the hydrophilic vehicle, whereas laurocapram and oleic acid combination decreased the permeation. The permeation parameters expressed in Table 5 demonstrate that the combination with nerolidol yielded comparable parameters to those of the vehicle without the enhancer (control) (*p* > 0.05). Laurocapram and oleic acid, on the other hand, significantly reduced the permeation of CBD (*p* < 0.05). Their *J_ss_* and *P_s_* values decreased approximately 0.4 and 0.7 fold for laurocapram and oleic acid, respectively, when compared to the vehicle without an enhancer. This was due to the decreased *K* values with the addition of laurocapram and oleic acid (*p* < 0.05). Interestingly, in the case of laurocapram, *T_lag_* was shortened, which was related to an increase in the *D_ss_* value (*p* < 0.05).

Figure 4B illustrates the deposition of CBD after permeation using the 25:75 PG: DEGEE vehicle, with and without a combined enhancer. CBD deposition from the vehicle with a combined enhancer was in the range of 374.3 ± 67.9 to 681.9 ± 86.1 μg·cm^−2^, which was 0.4–0.7 fold lower than that of the control (*p* < 0.05). The CBD deposition amount was in the order of vehicle without enhancer > oleic acid > laurocapram~~nerolidol (*p* < 0.05). The vehicle uptake capacity results (Table 5) indicated that the vehicle uptake capacity was improved with the addition of nerolidol, while laurocapram and oleic acid exhibited insignificant and lessening effects on the uptake capacity, respectively. It seems that these vehicle uptake capacity values were not associated with the CBD permeation and deposition results.

**Figure 5:**
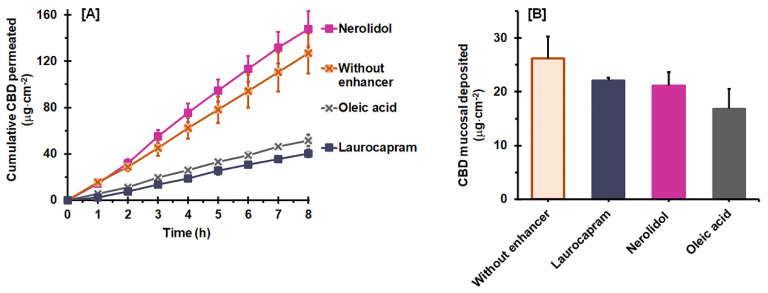
Effect of combined enhancers on ex vivo CBD permeation (**A**) and deposition (**B**) with the 25:75 DEGEE:MCT binary vehicles (mean ± SD, *n* = 4).

Figure 5A presents the influence of the combined enhancers on the mucosal permeation behavior of CBD with 25:75 DEGEE:MCT binary vehicle. It was found that laurocapram and oleic acid showed strong retardation effects, while nerolidol exhibited a slight enhancing effect on CBD permeation. The corresponding permeation parameters of CBD, as presented in Table 6, indicated a significant reduction in *J_ss_*, by approximately 0.3 and 0.4 fold, with laurocapram and oleic acid, respectively. These reductions resulted from decreases in the *P_s_* values, likely related to the decrease in *D_ss_* and/or *K*. Interestingly, in the case of laurocapram, *T_lag_* was lengthened, which was related to the decreased *D_ss_* value (*p* < 0.05). Similar to the hydrophilic vehicle, the combination with nerolidol exhibited an insignificant influence on the CBD permeation from DEGEE:MCT as all of its permeation parameters were comparable to those of the control (*p* > 0.05).

The effects of the combined enhancers on the deposition of CBD after permeation from a lipophilic vehicle are presented in Figure 5B. The vehicles with combined enhancers yielded CBD deposition in the range of 16.8 ± 3.7 to 22.1 ± 0.5 μg·cm^−2^. No significant differences were found between the different combined enhancers investigated (*p* > 0.05). However, when compared with the vehicle without an enhancer, it can be clearly seen that the CBD deposition decreased significantly with the addition of enhancer, with an approximately 0.6–0.8 fold decrease (*p* < 0.05). The vehicle uptake capacity of the 25:75 DEGEE:MCT vehicle was reduced with oleic acid addition, while laurocapram and nerolidol exhibited insignificant effects (Table 6).

Laurocapram is a highly lipophilic permeation enhancer with log P value of 6.2 [45]. Nerolidol is noncyclic sesquiterpene alcohol with calculated log P of 5.68, belonging to the terpene class of chemical enhancers. Terpenes of natural origin are generally recognized as safe (GRAS) components with rather low and reversible irritation [10,63]. Oleic acid is a long-chain unsaturated fatty acid (C18:1) with log P of 7.64, which has been shown to increase the permeation of lipophilic permeant across buccal mucosa via the transcellular pathway [10,21,45,64]. Oleic acid has recently been demonstrated to be a useful permeation enhancer for skin delivery of CBD. A higher permeation flux with shortened lag time was achieved through the combination of oleic acid. The cumulative permeated CBD at 4 h from neat PG vehicle has been improved by 3.9 fold through the addition of 5% oleic acid [39]. Previously, Touitou and Fabin [34] have reported that oleic acid significantly enhanced the percutaneous permeation of Δ^8^-tetrahydrocannabinol through hairless mouse skin with PG:ethanol (50:50) solutions. The presence of 3–10% oleic acid in PG:ethanol vehicle yielded the greatest enhancement, with 7-fold increment in the permeation flux and 6-fold increment in the permeability coefficient.

Laurocapram, oleic acid, and nerolidol have been shown to be promising enhancers for different permeants, enhancing permeation into and across biological membranes. Their mechanism of action has been reported to involve lipid structure perturbation, creating a region of fluidity in intercellular lipids and improving permeant diffusivity [10,11,21]. With regard to their physico-chemical properties and mechanism of permeation enhancement, laurocapram, oleic acid, and nerolidol were hypothesized to enhance the mucosal permeation of CBD when combined with the considered vehicles. Unfortunately, insignificant or retardation effects were instead observed, which may be attributed to their highly lipophilic nature, especially laurocapram and oleic acid. The addition of these enhancers into the vehicles would boost the vehicle lipophilicity and, therefore, the affinity of CBD to the vehicle, thus lessening the CBD partitioning into the mucosa. This is in line with previous reports. It has been demonstrated that the solubility (and, thus, partition coefficients) of physostigmine in PG and mineral oil, as vehicles, increased with the addition of fatty acid. This effect was more pronounced with long chain fatty acids, such as oleic acid and linoleic acid (C18:2) [65]. Nicolazzo et al. [11] have reported that the addition of 5% laurocapram into a marketed formulation, Kenalog in Orabase, exhibited no beneficial effect on triamcinolone acetonide permeation through and deposition in the porcine buccal mucosa. On the other hand, pre-treatment of the buccal mucosa with 5% laurocapram in ethanol significantly enhanced both the permeation and deposition of triamcinolone acetonide. Hansen et al. [66] have stated that the impact of 5% oleic acid on the cell membrane of the porcine buccal mucosa was probably insufficient to compensate for the effect of PG on the partitioning of diazepam between vehicle and epithelium. Junaid et al. [39] have recently reported the retardation effect of terpenes on the percutaneous permeation of CBD. Terpene addition, in the form of 5% essential oils—namely, peppermint oil, eucalyptus oil, and lavender oil—into the PG vehicle significantly diminished the permeation of CBD.

## 4. Conclusions

This is the first study emphasizing the ex vivo mucosal permeation and deposition of CBD under fixed-dose conditions considering a range of neat lipophilic and hydrophilic vehicles, as well as binary vehicles in hydrophilic and hydrophilic–lipophilic systems. Of the neat vehicles examined, PG and DEGEE exhibited the greatest enhancement effects; notably, these were the vehicles in which CBD was the least soluble. The binary system of these two vehicles further boosted the permeation and deposition of CBD, where the 25:75 PG:DEGEE system provided the greatest value amongst all the investigated systems. This binary vehicle could be considered a good vehicle for the mucosal delivery of CBD.

To the best of our knowledge, this is the first study to examine both mucosal permeation flux and deposition of CBD from MCT, the vehicle commonly found in CBD oil, as well as hemp seed oil. The poor ability of MCT to deliver CBD across the mucosa was revealed. The mucosal permeation and deposition of CBD from the MCT vehicle could be modified through addition of a hydrophilic additive; namely, DEGEE. It is also interesting to note that the addition of lipophilic enhancers—namely, laurocapram, oleic acid, and nerolidol—to either hydrophilic or lipophilic vehicles significantly diminished the mucosal permeation and deposition of CBD. These phenomena were associated with the lipophilicity of the vehicle systems. The corresponding permeation parameters further highlighted the important role of CBD solubility and affinity to the vehicle, which directly influences its partitioning into the mucosa. When a fixed concentration of CBD solution was applied, as in this investigation or the case of commercial product application, the thermodynamic activity was not maintained as equal. The permeation of an enhancer, as well as the alteration in mucosa membrane, may not be sufficient to compensate for the thermodynamic activity alteration of the vehicle by the combined enhancer. The results obtained in this study suggests that, in terms of permeation and deposition ability, hydrophilic liquids and additives are the composition of choice for cannabinoid formulations intended for transmucosal delivery. This study contributes necessary information for the design and development of CBD formulations intended for transmucosal administration. Additionally, the provided evidence could also be applied to other cannabinoids, due to their similar chemical structure and physicochemical properties.

## Figures and Tables

**Table 1:** Solubility of CBD in neat vehicles at 37 ± 0.5 °C.

Liquid Vehicles			CBD Solubility (mg/mL)
Types	Hansen Solubility Parameters (MPa^1/2^) *	Dielectric Constants **	
MCT	18.8	3.9	>1000 ***
DEGEE	21.4	14.1	598.0 ± 12.2
PG	29.2	32.1	514.0 ± 54.7
PEG 300	22.5	14.5	>1000 ***

Mean ± SD, *n* = 3. * Total Hansen solubility parameter: MCT, adapted from [29]; PEG 300, adapted from [30]; DEGEE, adapted from [31]. ** Dielectric constants values at 25 °C, adapted from [23,26,28]. *** A supersaturated CBD solution in MCT or PEG 300 was not achieved. The clear and transparent solution was obtained after mixing and 24-h incubation at 37 °C of 1 g CBD with 1 mL of vehicle.

**Table 2:** Vehicle uptake capacity of mucosa and ex vivo permeation parameters of CBD with neat vehicles.

Vehicles	Vehicle Uptake Capacity (%) ^1^	*J_ss_* (µg·cm^−2^ h^−1^) ^2^	*T_lag_* (h) ^2^	*Q*_8*h*_ (µg) ^2^	*P_s_* (×10^−3^ cm h^−1^) ^2^	*D_ss_* (×10^−3^ cm^2^ h^−1^) ^2^	*K* ^2^
MCT	12.2 ± 2.4 ^a^	0.89 ± 0.15 ^a^	2.17 ± 0.18 ^a^	10.78 ± 1.71 ^a^	0.036 ± 0.006 ^a^	0.307 ± 0.031 ^a^	0.006 ± 0.002 ^a^
DEGEE	7.8 ± 0.9 ^b^	163.01 ± 18.89 ^b^	2.09 ± 0.31 ^a^	2009.41 ± 230.92 ^b^	6.510 ± 0.754 ^b^	0.320 ± 0.043 ^a^	1.287 ± 0.162 ^b^
PG	30.9 ± 2.6 ^c^	179.81 ± 23.46 ^b^	1.72 ± 0.25 ^a^	2312.41 ± 380.39 ^b^	7.181 ± 0.937 ^b^	0.386 ± 0.066 ^a^	1.173 ± 0.151 ^b^
PEG 300	3.0 ± 0.1 ^d^	17.66 ± 3.12 ^a^	2.15 ± 0.10 ^a^	218.65 ± 35.89 ^a^	0.705 ± 0.125 ^a^	0.312 ± 0.012 ^a^	0.143 ± 0.024 ^a^

Mean ± SD, ^1^ *n* = 3, ^2^ *n* = 4. ^a–d^ Means in the same column without a common superscript letter are different (*p* < 0.05), as analyzed by one-way ANOVA and Tukey’s post-hoc test. *J_ss_* is the steady-state permeation flux (µg·cm^−2^ h^−1^) calculated within 2–8 h. *T_lag_* refers to lag time (h). *Q_8h_* is the cumulative CBD permeated at 8 h (µg). *P_s_* is the permeability coefficient of mucosa (cm h^−1^) and *D_ss_* is the apparent diffusivity through the mucosa (cm^2^ h^−1^). *K* refers to partition coefficient of CBD between mucosa and vehicle.

**Table 3:** Vehicle uptake capacity of mucosa and ex vivo permeation parameters of CBD with PG: DEGEE binary vehicles.

PG:DEGEE Ratio	Vehicle Uptake Capacity (%) ^1^	*J_ss_* (µg·cm^−2^ h^−1^) ^2^	*T_lag_* (h) ^2^	*Q*_8*h*_ (µg) ^2^	*P_s_* (×10^−3^ cm h^−1^) ^2^	*D_ss_* (×10^−3^ cm^2^ h^−1^) ^2^	*K* ^2^	ER
0:100	7.8 ± 0.9 ^a^	163.01 ± 18.89 ^a^	2.09 ± 0.31 ^a^	2009.41 ± 230.92 ^a^	6.510 ± 0.754 ^a^	0.320 ± 0.043 ^a^	1.287 ± 0.162 ^a^	1.0
25:75	13.3 ± 1.3 ^b^	244.74 ± 29.33 ^b^	1.84 ± 0.10 ^a^	3090.17 ± 407.06 ^b^	9.766 ± 1.170 ^b^	0.360 ± 0.012 ^a^	1.705 ± 0.143 ^b^	1.5
50:50	12.1 ± 1.2 ^b^	263.39 ± 46.68 ^b^	1.95 ± 0.05 ^a^	3272.40 ± 587.80 ^b^	10.494 ± 1.860 ^b^	0.330 ± 0.004 ^a^	1.980 ± 0.377 ^b^	1.6
100:0	30.9 ± 2.6 ^c^	179.81 ± 23.46 ^b^	1.72 ± 0.25 ^a^	2312.41 ± 380.39 ^b^	7.181 ± 0.937 ^b^	0.386 ± 0.066 ^a^	1.173 ± 0.151 ^b^	N/A

Mean ± SD, ^1^ *n* = 3, ^2^ *n* = 4. ^a–c^ Means in the same column without a common superscript letter are different (*p* < 0.05), as analyzed by one-way ANOVA and Tukey’s post-hoc test. N/A, not applicable. *J_ss_* is the steady-state permeation flux (µg·cm^−2^ h^−1^) calculated within 2–8 h. *T_lag_* refers to lag time (h). *Q_8h_* is the cumulative CBD permeated at 8 h (µg). *P_s_* is permeability coefficient of mucosa (cm h^−1^) and *D_ss_* is the apparent diffusivity through mucosa (cm^2^ h^−1^). *K* refers to partition coefficient of CBD between mucosa and vehicle. ER refers to the enhancement ratio of the permeation flux of PG:DEGEE vehicles as compared to neat DEGEE.

**Table 4:** Vehicle uptake capacity of mucosa and ex vivo permeation parameters of CBD with DEGEE:MCT binary vehicles.

DEGEE:MCT Ratio	Vehicle Uptake Capacity (%) ^1^	*J_ss_* (µg·cm^−2^ h^−1^) ^2^	*T_lag_* (h) ^2^	*Q*_8*h*_ (µg) ^2^	*P_s_* (×10^−3^ cm h^−1^) ^2^	*D_ss_* (×10^−3^ cm^2^ h^−1^) ^2^	*K* ^2^	ER
0:100	12.2 ± 2.4 ^a^	0.89 ± 0.15 ^a^	2.17 ± 0.18 ^a^	10.78 ± 1.71 ^a^	0.036 ± 0.006 ^a^	0.307 ± 0.031 ^a^	0.006 ± 0.002 ^a^	1.0
25:75	14.4 ± 1.1 ^a^	16.34 ± 2.40 ^b^	0.23 ± 0.05 ^b^	254.95 ± 35.26 ^b^	0.651 ± 0.095 ^b^	2.984 ± 0.835 ^b^	0.014 ± 0.004 ^b^	17.7
50:50	8.6 ± 0.9 ^b^	38.84 ± 3.63 ^c^	0.25 ± 0.05 ^b^	583.04 ± 118.71 ^c^	1.551 ± 0.145 ^c^	2.623 ± 0.425 ^b^	0.037 ± 0.004 ^c^	43.8
75:25	8.1 ± 0.8 ^b^	9.48 ± 1.59 ^d^	0.26 ± 0.07 ^b^	147.13 ± 25.01 ^b^	0.379 ± 0.064 ^d^	2.716 ± 0.728 ^b^	0.009 ± 0.002 ^a^	10.9
100:0	7.8 ± 0.9	163.01 ± 18.89	2.09 ± 0.31	2009.41 ± 230.92	6.510 ± 0.754	0.320 ± 0.043	1.287 ± 0.162	N/A

Mean ± SD, ^1^ *n* = 3, ^2^ *n* = 4. ^a–d^ Means in the same column without a common superscript letter are different (*p* < 0.05), as analyzed by one-way ANOVA and Tukey’s post-hoc test. N/A, not applicable. *J_ss_* is the steady-state permeation flux (µg·cm^−2^ h^−1^) calculated within 2–8 h. *T_lag_* refers to lag time (h). *Q_8h_* is the cumulative CBD permeated at 8 h (µg). *P_s_* is permeability coefficient of mucosa (cm h^−1^) and *D_ss_* is the apparent diffusivity through mucosa (cm^2^ h^−1^). *K* refers to partition coefficient of CBD between mucosa and vehicle. ER refers to the enhancement ratio of the permeation flux of DEGEE:MCT vehicles as compared to neat MCT.

**Table 5:** Effect of combined enhancers on vehicle uptake capacity of mucosa and ex vivo permeation parameters of CBD with PG:DEGEE system.

Vehicles	Vehicle Uptake Capacity (%) ^1^	*J_ss_* (µg·cm^−2^ h^−1^) ^2^	*T_lag_* (h) ^2^	*Q*_8*h*_ (µg) ^2^	*P_s_* (×10^−3^ cm h^−1^) ^2^	*D_ss_* (×10^−3^ cm^2^ h^−1^) ^2^	*K* ^2^	ER
Without enhancer	13.3 ± 1.3 ^a^	244.74 ± 29.33 ^a^	1.84 ± 0.10 ^a^	3090.17 ± 407.06 ^a^	9.766 ± 1.170 ^a^	0.360 ± 0.012 ^a^	1.705 ± 0.143 ^a^	1.0
Laurocapram	11.9 ± 1.1 ^a^	98.60 ± 12.41 ^b^	0.46 ± 0.17 ^b^	1498.15 ± 218.21 ^b^	3.925 ± 0.494 ^b^	1.627 ± 0.739 ^b^	0.168 ± 0.052 ^b^	0.4
Oleic acid	8.6 ± 1.2 ^b^	159.39 ± 15.59 ^c^	1.66 ± 0.17 ^a^	2057.66 ± 232.81 ^b^	6.376 ± 0.624 ^c^	0.408 ± 0.050 ^a^	0.997 ± 0.093 ^c^	0.7
Nerolidol	14.1 ± 0.9 ^a^	224.14 ± 21.87 ^a^	1.70 ± 0.15 ^a^	2905.16 ± 274.28 ^a^	8.923 ± 0.871 ^a^	0.384 ± 0.026 ^a^	1.457 ± 0.178 ^d^	0.9

Mean ± SD, ^1^ *n* = 3, ^2^ *n* = 4. ^a–d^ Means in the same column without a common superscript letter are different (*p* < 0.05), as analyzed by one-way ANOVA and Tukey’s post-hoct test. *J_ss_* is the steady-state permeation flux (µg·cm^−2^ h^−1^) calculated within 2–8 h. *T_lag_* refers to lag time (h). *Q_8h_* is the cumulative CBD permeated at 8 h (µg). *P_s_* is permeability coefficient of mucosa (cm h^−1^) and *D_ss_* is the apparent diffusivity through mucosa (cm^2^ h^−1^). *K* refers to partition coefficient of CBD between mucosa and vehicle. The combined enhancers (laurocapram, oleic acid and nerolidol) concentration investigated was 5% *w*/*v*. ER refers to the enhancement ratio of the permeation flux of vehicle with combined enhancer as compared to the control vehicle (without enhancer).

**Table 6:** Effect of combined enhancers on vehicle uptake capacity of mucosa and ex vivo permeation parameters of CBD with DEGEE:MCT system.

Vehicles	Vehicle Uptake Capacity (%) ^1^	*J_ss_* (µg·cm^−2^ h^−1^) ^2^	*T_lag_* (h) ^2^	*Q*_8*h*_ (µg) ^2^	*P_s_* (×10^−3^ cm·h^−1^) ^2^	*D_ss_* (×10^−3^ cm^2^ h^−1^) ^2^	*K* ^2^	ER
Without enhancer	14.4 ± 1.1 ^a^	16.34 ± 2.40 ^a^	0.23 ± 0.05 ^a^	254.95 ± 35.26 ^a^	0.651 ± 0.095 ^a^	2.984 ± 0.835 ^a^	0.014 ± 0.004 ^a^	1.0
Laurocapram	15.1 ± 0.7 ^a^	5.51 ± 0.54 ^b^	0.54 ± 0.18 ^b^	81.13 ± 6.82 ^b^	0.220 ± 0.022 ^b^	1.332 ± 0.562 ^b^	0.012 ± 0.002 ^a^	0.3
Oleic acid	13.6 ± 0.8 ^a^	6.71 ± 0.45 ^b^	0.19 ± 0.04 ^a^	103.60 ± 10.11 ^b^	0.268 ± 0.018 ^b^	3.727 ± 0.905 ^a^	0.005 ± 0.001 ^b^	0.4
Nerolidol	11.0 ± 1.2 ^b^	19.29 ± 1.95 ^a^	0.19 ± 0.05 ^a^	297.53 ± 30.64 ^a^	0.770 ± 0.078 ^a^	3.610 ± 0.589 ^a^	0.014 ± 0.003 ^a^	1.2

Mean ± SD, ^1^ *n* = 3, ^2^ *n* = 4. ^a,b^ Means in the same column without a common superscript letter are different (*p* < 0.05), as analyzed by one-way ANOVA and Tukey’s post-hoct test. *J_ss_* is the steady-state permeation flux (µg·cm^−2^ h^−1^) calculated within 2–8 h. *T_lag_* refers to lag time (h). *Q_8h_* is the cumulative CBD permeated at 8 h (µg). *P_s_* is permeability coefficient of mucosa (cm h^−1^) and *D_ss_* is the apparent diffusivity through mucosa (cm^2^ h^−1^). *K* refers to partition coefficient of CBD between mucosa and vehicle. The combined enhancers (laurocapram, oleic acid and nerolidol) concentration investigated was 5% *w*/*v*. ER refers to the enhancement ratio of the permeation flux of vehicle with combined enhancer as compared to the control vehicle (without enhancer).

## Data Availability

Data available on request.
